# Left ventricular remodeling following aortic root and ascending aneurysm repair

**DOI:** 10.3389/fcvm.2022.944786

**Published:** 2022-10-25

**Authors:** Ignas B. Houben, Angel K. Y. Chu, Bo Yang, Karen M. Kim, Shinichi Fukuhara, Joost A. van Herwaarden, Frans L. Moll, David A. Nordsletten, C. Alberto Figueroa, Nicholas S. Burris, Himanshu J. Patel

**Affiliations:** ^1^Department of Cardiac Surgery, University of Michigan, Ann Arbor, MI, United States; ^2^Department of Vascular Surgery, University Medical Center Utrecht, Utrecht, Netherlands; ^3^Department of Computational Medicine and Bioinformatics, University of Michigan, Ann Arbor, MI, United States; ^4^Department of Biomedical Engineering, University of Michigan, Ann Arbor, MI, United States; ^5^Department of Vascular Surgery, University of Michigan, Ann Arbor, MI, United States; ^6^Department of Radiology, University of Michigan, Ann Arbor, MI, United States

**Keywords:** ascending aorta, ventricular remodeling, computed tomography angiogram (CTA), aortic repair, aortic aneurysm (thoracic)

## Abstract

**Objective:**

Adverse left ventricular remodeling due to a mismatch between stiffness of native aortic tissue and current polyester grafts may be under-recognized. This study was conducted to evaluate the impact of proximal aortic replacement on adverse remodeling of the left ventricle.

**Materials and methods:**

All aortic root and ascending aortic aneurysm patients were identified (*n* = 2,001, 2006–2019). The study cohort consisted of a subset of patients (*n* = 98) with two or more electrocardiogram (ECG)-gated CT angiograms, but without concomitant aortic valve disease or bicuspid aortic valve, connective tissue disease, acute aortic syndrome or prior history of aortic repair or mitral valve surgery. LV myocardial mass was measured from CT data and indexed to body surface area (LVMI). The study cohort was divided into a surgery group (*n* = 47) and a control group; optimal medical therapy group (OMT, *n* = 51).

**Results:**

The mean age was 60 ± 11 years (80% male). Beta-blocker use was significantly more frequent in the surgery group (89 vs. 57%, *p* < 0.001), whereas, all other antihypertensive drugs were more frequent in the OMT group. The average follow-up was 9.1 ± 4.0 months for the surgery group and 13.7 ± 6.3 months for the OMT group. Average LVMI at baseline was similar in both groups (*p* = 0.934). LVMI increased significantly in the surgery group compared to the OMT group (3.7 ± 4.1 vs. 0.6 ± 4.4 g/m^2^, *p* = 0.001). Surgery, baseline LVMI, age, and sex were found to be independent predictors of LVMI increased on multivariable analysis.

**Conclusion:**

Proximal aortic repair with stiff polyester grafts was associated with increased LV mass in the first-year post-operative and may promote long-term adverse cardiac remodeling. Further studies should be considered to evaluate the competing effects of aortic aneurysm related mortality against risks of long-term graft induced aortic stiffening and the potential implications on current size thresholds for intervention.

## Introduction

The most frequent location of thoracic aortic aneurysms (TAA) is the ascending aorta (1). While optimal medical therapy (OMT) focused on aggressive blood pressure management is thought to prevent acute complications (e.g., dissection and rupture), definitive management of TAA involves open surgical repair (2). The proximal aorta is the most compliant segment of the aorta and is a large contributor to the Windkessel effect (i.e., the elastic reservoir function of large vessels) (3). This effect allows for a reduction in left ventricular impedance and continued forward flow in diastole. It has been previously reported that dilation of the ascending aorta is correlated to an increase in left ventricular (LV) mass and a change in left ventricular function (4). This observation is likely due to altered ventricular-arterial coupling (VAC) in TAA secondary to loss of elastin and collagen remodeling, resulting in aortic stiffening and dilation, and subsequent increase in LV afterload (4, 5).

Current guidelines recommend that ascending TAA undergo repair when maximal aortic diameter reaches threshold sizes—typically 50–55 mm—to prevent acute complications (2, 6). The gold standard for treatment of ascending TAA is open surgical repair with synthetic graft material, commonly made of polyester. These synthetic grafts have a radial stiffness at normal blood pressure values that is approximately 50 times higher than that of native aortic tissue, and can potentially increase left ventricular afterload with subsequent LV hypertrophy and remodeling (7–10). van Bakel et al. (11) suggested a clinically measurable effect of stiff synthetic endograft on the LV remodeling in a small cohort of eight patients after descending aortic aneurysm repair. The effects of stiff graft on the LV may be even larger in the ascending aorta owing its more central location and comparatively higher degree elastance. In this study, we aimed to evaluate the effects of synthetic graft replacement of the ascending aorta on left ventricular mass in patients undergoing surgical repair for ascending TAA. We hypothesized that the LV mass increase is larger in patients undergoing synthetic graft replacement of the ascending aorta compared to patients with ascending TAA treated with OMT only.

## Materials and methods

Approval for this retrospective study was obtained from our local institutional review board (Protocol number HUM00169089, approved on 09/25/2019), the need for patient consent was waived.

### Patient selection

**FIGURE 1:**
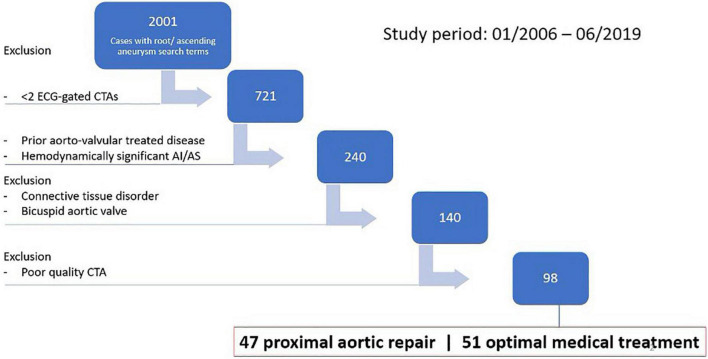
Inclusion and exclusion chart.

The Adult Cardiac Surgery database at the University of Michigan Frankel Cardiovascular Center was retrospectively queried for all patients presenting with aortic root and ascending aortic aneurysms between January 2006 and January 2019 (*n* = 2,001). Specifically, patients were identified *via* the Society of Thoracic Surgeons (STS) database and our institutional Electronic Medical Record Search Engine (EMERSE) (12). For information on our specific terms please refer to the Supplementary material. Patients were included if they had at least two electrocardiographically (ECG)-gated computed tomography angiography (CTA) scans and an aortic root or ascending aortic dilation with a diameter ≥40 mm. Patients were excluded for having (1) a history of hemodynamically significant (moderate or severe) aortic insufficiency (AI) or aortic stenosis (AS), (2) bicuspid aortic valve (BAV), (3) connective tissue disease (CTD), (4) any prior acute aortic syndrome or thoracic aortic repair, (5) prior aortic or mitral valve repair or replacement (AVR or MVR), (6) index repair that included AVR or MVR, or (7) a CTA follow-up interval of <4 months. After exclusion, 140 patients were selected for further review, after which an additional 42 patients were excluded based on poor quality CTA data preventing accurate segmentation of the LV myocardium (Figure 1). The population was divided into a treatment group, receiving open valve-sparing root repair and/or ascending repair in addition to OMT, and a control group, receiving OMT only. These will be further referred to as the surgery group and the OMT groups, respectively.

### Clinical data

Clinical data was gathered from the electronic medical records including: brachial blood pressures at baseline and follow-up during clinic visits, maximal proximal (root or ascending) aortic diameter at baseline and follow-up from radiologic reports, antihypertensive drugs (AHD) at baseline and follow-up, body surface area (BSA) at baseline and follow-up, and history of hypertension, dyslipidemia, diabetes mellitus, coronary artery disease, and tobacco use. For AHD, reports on five type of drugs were collected: diuretics, angiotensin-converting enzyme (ACE)-inhibitors, calcium-channel blockers (CCB), angiotensin-receptor blockers (ARB), and beta-blockers (BB).

### Imaging data

**FIGURE 2:**
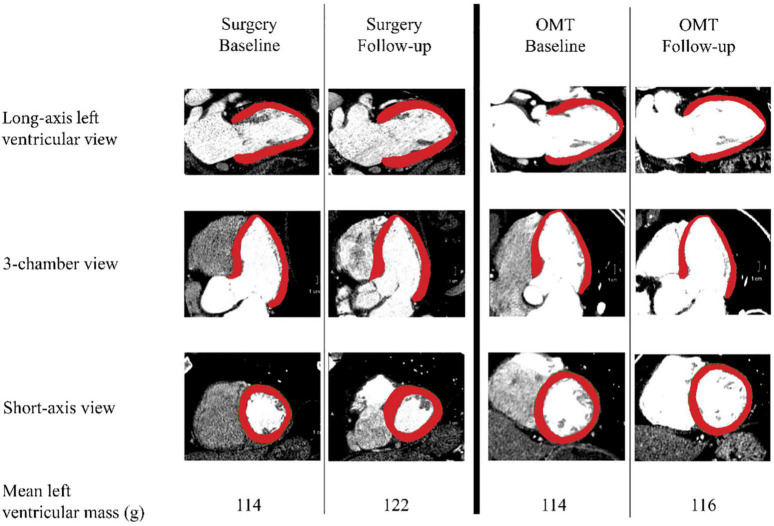
Measurement methods. Left ventricular mass was calculated by multiplying myocardial volume multiplying by estimated tissue density of 1.055 gr/ml. Surgery and the optimal medical treatment (OMT) group are divided by the thick black vertical line in the middle. Per group, the two columns show an example of a baseline and a follow-up measurement in three different views. The bottom row depicts the mean values of the left ventricular masses in grams for each measurement point per group.

Computed tomography angiogram examinations were acquired on a multi-slice scanner after intravenous injection of 120 ml iopamidol intravenous contrast (Isoview 370, Bracco Diagnostics, Milan, Italy) and prospective reconstruction was performed (75% of the R-R interval). The CTA image data were analyzed using automatic image processing tools in the software package Vitrea Core (Vital Images Inc., Product Version 6.9.1, Minnetonka, MN, USA). All measurements of myocardial mass were performed by two observers, IBH and AKYC, using a semi-automated delineation of the myocardial volume between the endo- and epicardial borders, multiplied by a tissue density of 1.055 gr/ml (Figure 2). LV myocardial mass was indexed to body surface area, yielding left ventricular mass index (LVMI) in g/m^2^. In the surgery group, the baseline scan was defined as the pre-operative CTA closest to the date of surgery.

A subgroup of 40 randomly selected CTAs were analyzed in duplicate by each observer to determine intra- and interobserver agreement of LV mass measurements. A 2-week interval between intra-observed measurements was employed to minimize recall bias. For these results, please refer to the Supplementary material.

### Statistical analysis

Continuous data, determined to be normally distributed by Shapiro-Wilk normality test, was presented as mean ± SD; otherwise, non-parametric, continuous data was presented as median (IQR). For categorial data, the frequency and percentage were summarized in Tables 1, 2. If a variable had more than 10% missing data, the amount missing was specified in the tables. Intra- and interobserver agreement were analyzed using a concordance correlation coefficient (CCC) and graphically depicted using Bland-Altman plots with limits of agreement (13). CCC values <0.90 were considered “poor agreement,” between 0.90 and 0.95 “moderate,” 0.95–0.99 “substantial,” and >0.99 “almost perfect” as described by Lin et al. (14). To evaluate the differences between the means (or medians) in clinical and imaging data of the case and control groups, independent *t*-test was used for the normally-distributed continuous data, whereas Mann-Whitney U test was used for the non-normally-distributed data. Pearson’s chi-squared test was applied for the categorical data, while Fisher’s exact test was run for categorical data with small-sized samples (*n* ≤ 5). Multiple linear regression analysis was performed to identify independent predictors of change in LVMI amongst a group of potential confounders. Statistical significance was defined as *p* < 0.05. Analysis of the data was performed using SPSS Statistics 26 (IBM, Armonk, NY, USA) and R3.6.2 [R Core Team, (15)].

**TABLE 1:** Demographics and comorbidities.

Patient characteristic	Overall	Surgery	OMT	*P*-value
		
	(*n* = 98)	(*n* = 47)	(*n* = 51)	
Age, years ± SD	59.9 ± 10.5	60.1 ± 10.5	59.7 ± 10.5	0.821
Sex, male (%)	78 (80)	38 (81)	40 (78)	0.963
BSA, m^2^ ± SD	2.09 ± 0.25	2.09 ± 0.21	2.09 ± 0.29	0.915
BMI, median (IQR)	28.7 (26.2–31.3)	28.9 (26.2–30.7)	28.3 (26.2–30.4)	0.548^∧^
Aortic root dilation only, *n* (%)*	20 (20)	10 (21)	10 (20)	0.838
Hypertension, *n* (%)	66 (68)	34 (72)	32 (63)	0.312
Coronary artery disease, *n* (%)	17 (17)	12 (26)	5 (10)	0.074
Diabetes mellitus, *n* (%)	7 (7)	4 (9)	3 (6)	0.717^†^
Chronic kidney disease, *n* (%)	5 (5)	2 (4)	3 (6)	0.715^†^
Tobacco use, *n* (%)	38 (39)	18 (38)	20 (39)	0.926
Dyslipidemia, *n* (%)	44 (45)	21 (45)	23 (45)	0.967
Prior cardiovascular repair, *n* (%)	5 (5)	4 (9)	1 (2)	0.191^†^
Diuretic use, *n* (%)	16 (16)	4 (9)	12 (24)	0.044
ACE-inhibitor use, *n* (%)	22 (22)	4 (9)	18 (35)	0.002
Calcium-channel blocker use, *n* (%)	12 (12)	2 (4)	10 (20)	0.036
Angiotensin-receptor blocker use, *n* (%)	14 (14)	2 (4)	12 (24)	0.006
Beta-blocker use	71 (72)	42 (89)	29 (57)	<0.001
Pulse pressure, mmHg ± IQR	49 (40–59)	50.5 (40.8–58.8)	48.5 (40.2–58.8)	0.613^∧^
Systolic blood pressure, mmHg ± SD	130 ± 17	133 ± 16	128 ± 17	0.115
Diastolic blood pressure, mmHg ± SD	79 ± 9	81 ± 10	77 ± 8	0.046

*No ascending dilation involvement reported between the sinotubular junction and the innominate artery. ^†^Fisher’s exact test; ^∧^Mann-Whitney U test. IQR, interquartile range; SD, standard deviation; BSA, body surface area; BMI, body mass index; ACE-inhibitor, angiotensin-converting enzyme inhibitor; LVM, left ventricular mass; LVMI, left ventricular mass index; LVMI Change, the difference between left ventricular mass index at baseline and during follow-up. Anthropometric and relevant clinical data was available for 98 patients. Baseline demographic, medical history, antihypertensive therapy, clinical characteristics and pressures are presented in Table 1. *P*-values were calculated with *t*, MWW, or χ^2^ tests to compare baseline values in the participants who underwent valve-sparing root replacement with those who did not undergo valve-sparing root replacement.

**TABLE 2:** Imaging characteristics.

Patient characteristic	Overall	Surgery	OMT	*P*-value
		
	(*n* = 98)	(*n* = 47)	(*n* = 51)	
Mild aortic insufficiency, *n* (%)*	28 (29)	17 (37)	11 (22)	0.11
**Baseline aortic diameter, mm median (IQR):**				
Root	45 (42–50)	49 (45–52.5)	43 (39.7–46.4)	<0.001^∧^
Sinotubular junction	40 (37–43)	42 (39–44.8)	39 (36–41.3)	<0.001^∧^
Mid-ascending	44.8 (40.2–47)	45 (41.6–49.1)	44 (40–46)	0.040^∧^
Overall maximum aortic diameter	45 (41.1–47.7)	46 (43–50)	44 (40.4–46)	0.002^∧^
CT scan interval, days median (IQR)	340 (210–392)	242 (188–296)	368 (344–472)	<0.001^∧^
**Left ventricular parameters:**				
LVM, g ± SD	114 ± 27	114 ± 25	114 ± 30.0	0.956
LVMI at baseline, g/m^2^ ± SD	54.3 ± 9.8	54.4 ± 9.5	54.2 ± 10.2	0.934
LVMI change, g/m^2^ ± SD	2.08 ± 4.5	3.67 ± 4.1	0.62 ± 4.4	<0.001

*Significant aortic insufficiency was excluded from the dataset, so this group constitutes only patients with mild, minimal/no insufficiency. ^∧^Mann-Whitney U test. IQR, interquartile range; SD, standard deviation; CT, computed tomography; LVM, left ventricular mass; LVMI, left ventricular mass index. A table containing the radiographic characteristics describe aortic valve insufficiency and proximal aortic diameter based on radiographic reports. Additionally, the CT scan interval is described, as well as the measurements of the left ventricular myocardial tissue. Between groups, a significant difference was found for the proximal diameters and the average interval between the two CT scans.

## Results

### Baseline characteristics

Ninety eight patients were included for analysis, 47 in the surgical group and 51 in the OMT group. Overall, 78 patients (80%) were male and mean age was 60 ± 11 years. No significant differences between groups were found in sex, age, hypertension, dyslipidemia, diabetes mellitus, coronary artery disease, and tobacco use. BB use was more frequent in the surgery group (89 vs. 57%, *p* < 0.001) whereas ACE (35 vs. 9%, *p* = 0.002), ARB (24 vs. 4%, *p* = 0.006), diuretic (24 vs. 9%, *p* = 0.002) and CCB (20 vs. 4%, *p* = 0.044) use were more frequent in the OMT group. There was a small but statistically significant difference in baseline diastolic pressure between the surgery group and the OMT group (81 vs. 77 mmHg, *p* = 0.046). Patient characteristics are detailed in Table 1. The distribution of ascending aortic dilation (diameter > 40 mm) in our population was as follows: 20% isolated aortic root dilation, 34% isolated mid-ascending aortic dilation, and 46% both aortic root and ascending aortic dilation. The surgery group had significantly higher baseline diameters at the levels of the root, sinotubular junction, and the mid ascending aorta. No differences were found in baseline left ventricular mass between the surgery and the OMT group (54.5 ± 9.5 vs. 54.2 ± 10.2 g/m^2^, *p* = 0.934). Aortic and LV imaging characteristics are detailed in Table 2.

### Follow-up characteristics

The CT follow-up interval for the surgery group was 9.1 ± 4.0 months, and for the OMT group was 13.7 ± 6.3 months, *p* < 0.001. The average aortic growth in the OMT group at aortic root level was 0.5 ± 1.6 mm and at the mid ascending aortic level it was 0.1 ± 1.2 mm.

**FIGURE 3:**
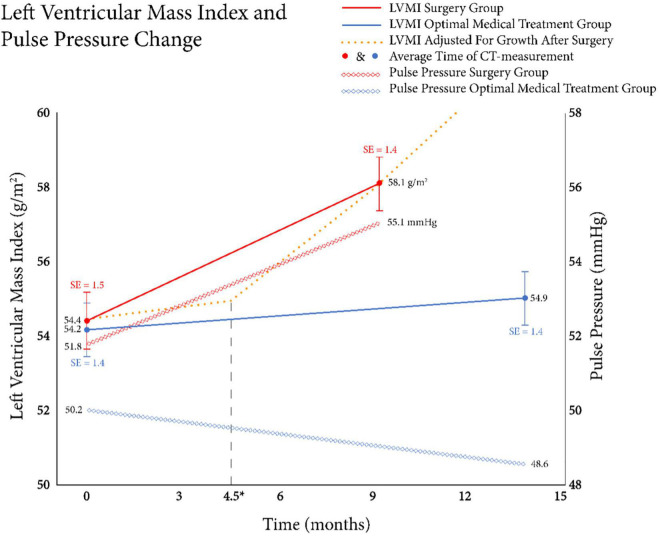
Relationship between left ventricular mass index (LVMI) change and pulse pressure change. The mean LVMI was shown in the left y-axis and the solid lines show the mean LVMI at the two measured time points for both the surgery + optimal medical treatment (OMT) group (red) and the OMT group (blue). The x-axis depicts the follow-up time in months. A significant difference in LVMI increase between groups was seen, respectively: 3.7 vs. 0.6 g/m^2^ (*p* = 0.001; Student’s *t*-test). Black dashed vertical line depicts the average time of the surgery after the baseline CT. Additionally, the average pulse pressure changes over time between groups were shown as the diamond shaped lines. The right y-axis depicts the pulse pressures in millimeters of mercury. Although an increase in pulse pressure was found in the surgery + OMT group (3.3 mmHg), where a decrease was found in the OMT group (−1.6 mmHg), the difference between groups was not significant (*p* = 0.063).

Left ventricular myocardial index increased significantly in the surgery group compared to the OMT group (3.7 ± 4.1 vs. 0.6 ± 4.4 g/m^2^, *p* = 0.001, Figure 3). There was a trend toward higher pulse pressure change at follow-up in the surgery group, but the difference was not statistically significant (3.3 ± 12.6 vs. −1.6 ± 12.6 mmHg, *p* = 0.063, Figure 3).

### Bivariate and multivariate analysis

Prior to conducting the multivariable analysis, we examined bivariate correlations between LVMI change and clinical and radiographic independent variables. Bivariate analyses were performed for our entire population (*n* = 98), revealing a weak positive correlation between male sex and LVMI change (R = 0.208, *p* = 0.039). Subsequently, bivariable correlation was examined for each treatment group. The effect of surgery on LVMI in a bivariate analysis was positive with a coefficient of 3.050 g/m^2^ (*p* = 0.001). In the surgery group, a weak positive correlation was seen between LVMI increase and age (R = 0.343, *p* = 0.018). In both the surgery group and the OMT group, CCB use was associated with a positive effect on LVMI change (surgery: +7.2 g/m^2^; OMT: +3.5 g/m^2^; *p* = 0.031). However, considering the low frequency of CCB and ARB use in the surgery group (2/47, 4%), these variables were not included in the multivariable analysis. Bivariate analyses are detailed in Table 3.

**TABLE 3:** Univariate regression analysis of left ventricular mass index (LVMI) change with baseline clinical parameters among treatment groups.

Patient characteristic	Overall		Surgery		OMT	
	(*n* = 98)		(*n* = 47)		(*n* = 51)	
			
	LVMI change, g/m^2^		LVMI change, g/m^2^		LVMI change, g/m^2^	
			
	Coefficient	*P*-value	Coefficient	*P*-value	Coefficient	*P*-value
Age, years	0.07	0.11	0.13	0.018	0.004	0.95
BMI	0.079	0.41	0.019	0.89	0.17	0.18
Pulse pressure change, mmHg	0.016	0.67	–0.024	0.62	0.005	0.92
Maximal aortic diameter, mm	0.028	0.74	–0.14	0.16	0.042	0.8
Baseline LVMI, g/m^2^	–0.081	0.083	–0.93	0.15	–0.074	0.23
CT interval, days	–0.001	0.64	–0.001	0.82	0.004	0.21
Sex, male	2.34	0.04	2.76	0.069	1.75	0.25
Diuretic at baseline use	0.84	0.49	4.065	0.035	0.57	0.7
ACE-inhibitor use	1.8	0.1	4.69	0.014	2.42	0.068
Calcium-channel blocker use	2.16	0.18	4.41	0.29	3.53	0.039
Angiotensin-receptor blocker use	–1.7	0.24	3.03	0.47	–0.77	0.63
Beta blocker use	0.3	0.77	–0.71	0.72	–1.3	0.31

BMI, body mass index; LVMI, left ventricular mass index; CT, computed tomography; ACE-inhibitor, angiotensin-converting enzyme inhibitor. Univariate linear regression results are presented for each variable to assess their effect on LVMI change. In the overall group, only male sex revealed a positive significant effect on LVMI change.

Multivariable analysis was conducted to identify independent predictors of LVMI change amongst the following predictors at baseline: surgical repair, age, sex, BMI, diuretic use, ACE-inhibitor use, BB use, maximal ascending aortic diameter, baseline LVMI and time-interval between CTA studies. On adjusted analysis we identified the following independent predictors of LVMI increase at follow-up: surgery (β = 4.903, *p* < 0.001), age (β = 0.132, *p* = 0.002), male sex (β = 3.720, *p* = 0.001), BMI (β = 0.240, *p* = 0.009), and baseline LVMI (β = −0.158, *p* = 0.001). Multiple linear regression results are shown in Table 4.

**TABLE 4:** Multivariate regression analysis with left ventricular mass index (LVMI) change as the dependent variable.

Characteristics	Coefficient	Robust standard error	*P*-value	95% Confidence interval
Age, years	0.132	0.042	0.002	0.048; 0.216
Sex, male	3.72	1.065	0.001	1.604; 5.837
BMI	0.24	0.089	0.009	0.062; 0.417
Diuretic use	1.967	1.102	0.078	−0.223; 4.158
ACE-inhibitor use	1.638	0.979	0.098	−0.308; 3.584
Beta-blocker use	–1.362	0.925	0.144	−3.201; 0.476
Maximal aortic diameter, mm	–0.163	0.085	0.058	−0.332; 0.006
Baseline left ventricular mass index, g/m^2^	–0.158	0.044	0.001	−0.246; -0.070
CT interval, days	0	0.002	0.953	−0.005; 0.005
Treatment, surgery	4.903	0.966	0	2.983; 6.824

## Discussion

We found that over the first year of post-operative imaging surveillance, the left ventricle undergoes a significant increase in mass following ascending aortic and/or aortic root graft repair, a relationship that persisted after adjustment for potential confounders. Furthermore, age, male sex, BMI, and baseline LVMI were also found to be significant predictors for LVMI increase in our cohort. Finally, although not statistically significant (*p* = 0.06), there was a trend toward increased pulse pressure observed in the surgery group, which may relate to the hemodynamic consequences of graft-induced increased proximal aortic stiffening. Finally, we demonstrated that our approach of using ECG gated CTA data to measure LV mass had low bias and acceptable levels of inter- and intra-rater reproducibility, supporting a recent study validating this method for evaluation of LVMI (16).

Stiffening of the proximal aorta is known to have a detrimental effect on cardiovascular health (17, 18). One of the direct adverse consequences of pathological central stiffening is an increase in afterload and altered ventricular-arterial coupling, promoting the development of left ventricular hypertrophy and increased risk of diastolic heart failure (4, 19, 20). Although previous animal studies have described left ventricular mass change following acute aortic stiffening (21, 22), the phenomenon is not as well-described in humans. Increased LVMI following central stiffening has been associated with an increased risk of heart failure (23). These studies, however, demonstrate the adverse effects of central arterial stiffening in a chronic process, where loss of compliance due to elastin degradation leads to systolic hypertension, widened pulse pressure, and further age-related arterial stiffening. Conversely, our study describes the consequences of an acute change of central artery stiffness related to synthetic graft implantation with a vastly higher order of magnitude of stiffening compared to that seen with aging. In the short term, the hemodynamics consequences of such a rapid change in proximal aortic stiffness may be significant, as suggested by our data and previously described in animal models (21, 22, 24).

Stiff polyester fabric grafts have been used to replace segments of the aorta for decades. The recent emergence of endovascular aortic repair (TEVAR) has been used to reduce the morbidity associated with open operative intervention; however, these metallic endografts also display stiffness properties many folds higher than seen with native aortic tissue. While aortic endografts are most commonly used to repair the descending thoracoabdominal aorta, which contributes much less to the aorta’s Windkessel function than the ascending aorta, a recent computational and imaging study from our group suggested that TEVAR may still result in an increase in LV mass due to its effects on aortic compliance (11). Clearly the benefits of repairing an aneurysmal segment of aorta, either by open or endovascular repair, greatly outweigh the risk of stiffness-induced adverse LV remodeling. However, our results suggest that such interventions may come with underappreciated consequences on the LV, which are measurable within the first post-operative year, but may be even more substantial over longer follow-up periods. We believe our results emphasize the need for aggressive blood pressure management in patients after graft replacement of the ascending aorta. Our results suggest a potential role for increased used of ACE-I/ARB medications after ascending aortic replacement given the favorable effects on these drugs on adverse cardiac remodeling (rates of ACE-I/ARB were low in our surgical group).

The positive correlation between age and LVMI increase also seen in the surgery group suggests that patients with advanced age may be at highest risk for adverse remodeling after open TAA repair. Age is known to be associated with a relative left ventricular mass increase, manifested as concentric hypertrophy, as a result of vascular stiffening (25, 26). Furthermore, age has also been associated with unfolding/lengthening of the aortic arch, decreased aortic distensibility and increased arch pulse wave velocity and central aortic pressures (25). Thus, the effects of graft-related aortic stiffening may be more pounced in older patients in whom native aortic stiffness is higher and thus possibly less able to buffer the deleterious effects of ascending aortic stiffening due to graft repair, however, these relationships warrant further study to clarify the mechanism of this observed association.

Additionally, we identified male sex as a predictor of LVMI increase in the regression analysis. Having a higher incidence of LVMI increase in male patients and in older patients has been previously described. Specifically, a large population study of 741 patients who underwent cardiac MRI demonstrated a high degree of concentric hypertrophy in males and may explain our observations (27). Lastly, the results of our multivariable suggest a potential negative effect of baseline LVMI on LVMI increase during follow-up. While the exact mechanism underlying this observation is unclear, it seems plausible that patients with higher degrees of LV hypertrophy at baseline may have less potential for further hypertrophy, supporting the theory of left ventricular hypertrophy reaching a plateau, a phenomenon which has been previously described (24).

### Limitations

There are several limitations to our study. First, our population was relatively modest in size (*n* < 100) and heterogeneous, with differences in follow-up intervals and antihypertensive medication that could lead to confounding, a limitation which is difficult to avoid given the retrospective nature of the study. We attempted to adjust for such group differences with multivariable analysis, however, our results should be considered hypothesis generating with further studies required to determine the independent effects of specific co-morbidities and medical therapies. Second, we used CTA based myocardial segmentation to measure changes in LV mass, an approach which is less commonly used compared to echocardiography or cardiac MRI, however, our results show comparable degrees of intra- and inter-rater agreement compared to cardiac MRI, which is generally considered the gold-standard for longitudinal LV mass measurements (28). Thirdly, our study did not employ techniques capable of directly quantifying the stiffness mismatch between graft material and native aortic tissue; however, given that the stiffness of aortic grafts has been estimated to be approximately 50 times that of native aortic tissue, we do believe that stiffness mismatches exist after ascending aortic repair. Finally, patients in both the surgery and OMT groups of our study had enlarged ascending aortas at baseline, and thus the additive effects of graft-induced stiffening may have be muted by pre-existing stiffening of the native aortic tissue; however, this scenario represents the clinical reality given that graft replacement of non-dilated ascending aorta is almost never indicated.

## Conclusion

Left ventricular mass increases within the first year after graft-replacement of the ascending aorta compared to patients with dilated ascending aortas who receive medical therapy only. This study represents one of the first attempts to investigate the effects of isolated ascending aortic replacement with stiff polyester fabric grafts on adverse LV remodeling using ECG-gated CTA data. Given that aortic surgery is performed for a life expectancy benefit, further studies should be considered to evaluate the competing effects of aortic aneurysm related mortality against risks of long-term graft induced aortic stiffening and the potential implications on current size thresholds for intervention. Finally, our results support the investigation of alternative materials for aortic replacement which are more compliant while still maintaining durability.

## Data availability statement

The original contributions presented in the study are included in the article/Supplementary material, further inquiries can be directed to NB, nburris@med.umich.edu.

## Ethics statement

The studies involving human participants were reviewed and approved by University of Michigan Institutional Review Board (Protocol number HUM00169089). Written informed consent for participation was not required for this study in accordance with the national legislation and the institutional requirements.

## Author contributions

IH, AC, and NB organized the database and performed the statistical analysis. IH wrote the first draft of the manuscript. IH, NB, HP, CF, and JH wrote sections of the manuscript. All authors contributed to conception, design of the study, and manuscript revision and read and approved the submitted version.

## Abbreviations

OMT: optimal medical treatment
LV: left ventricle
ECG: electrocardiogram
CTA: computed tomography angiography
AI: aortic insufficiency
AS: aortic stenosis
BAV: bicuspid aortic valve
CTD: connective tissue disease
AVR: aortic valve replacement
MVR: mitral valve replacement
BSA: body surface area
LVMI: left ventricular myocardial index
CCC: concordance correlation coefficient
AHD: antihypertensive drug
ACE: angiotensin-converting enzyme
CCB: calcium-channel blocker
ARB: angiotensin-receptor blocker
BB: beta-blocker.
