# Supplementary material for: Left ventricular remodeling following aortic root and ascending aneurysm repair
Source: Front Cardiovasc Med. 2022 Oct 25;9:944786. doi: 10.3389/fcvm.2022.944786 (PMC9640592; doi:10.3389/fcvm.2022.944786)
Supplement: Supplementary file 1 [file Data_Sheet_1.docx]

Left Ventricular Remodeling Following Aortic Root and Ascending Aneurysm Repair

Ignas B. Houben MD ^1,6^; Angel K.Y. Chu MS^2^; Bo Yang MD PhD^1^; Karen M. Kim MD^1^; Shinichi Fukuhara MD^1^; Joost A. van Herwaarden MD PhD^6^; Frans L. Moll MD PhD^6^; David A. Nordsletten PhD^1,3^; C. Alberto Figueroa PhD^3,4^; Nicholas S. Burris MD^5^*; Himanshu J. Patel MD^1^*.

**Supplementary material**

**I. Data Search**

We inquired the Society of Thoracic Surgeons (STS) database for all patients with ascending aortic and/or aortic root aneurysms having received surgery at the University of Michigan Medical Center.

The search terms used to inquire data from our Electronic Medical Record Search Engine (EMERSE) consisted of the following term bundles:

- “aort*” AND “aneurysm*” OR
- “aort*” AND “ascend*” AND “aneurysm*” OR
- “aort*” AND “root” AND “aneurysm*”

**II. Intra- interobserver agreement for LV mass measurements.**Our assessment of intra- and interobserver agreement on left ventricular volumetric measurement yielded a concordance correlation coefficient (CCC) of 0.998 in both intra- and interobserver assessment with a p-value <0.001, concluding in an almost perfect intra- and interobserver agreement. The mean difference was -0.51 ± 1.64 grams for observer 1 and 0.70 ± 2.31 for observer 2, leading to respective 95% confidence intervals of -3.72 to 2.70 and -3.83 to 5.23 (**Supplementary Figure 1**). For the interobserver analysis, the mean difference was 0.44 ± 1.44 grams with a repeatability coefficient of 2.82, leading to a 95% Confidence interval of -2.38 to 3.26 grams difference. Nearly all interobserver measurements revealed a 3 gram difference or less between observers in an average LV mass measurement rate of 112 grams in our subanalysis group.

**Supplementary Figure 1** – Intra-observer agreement Bland-Altman plot


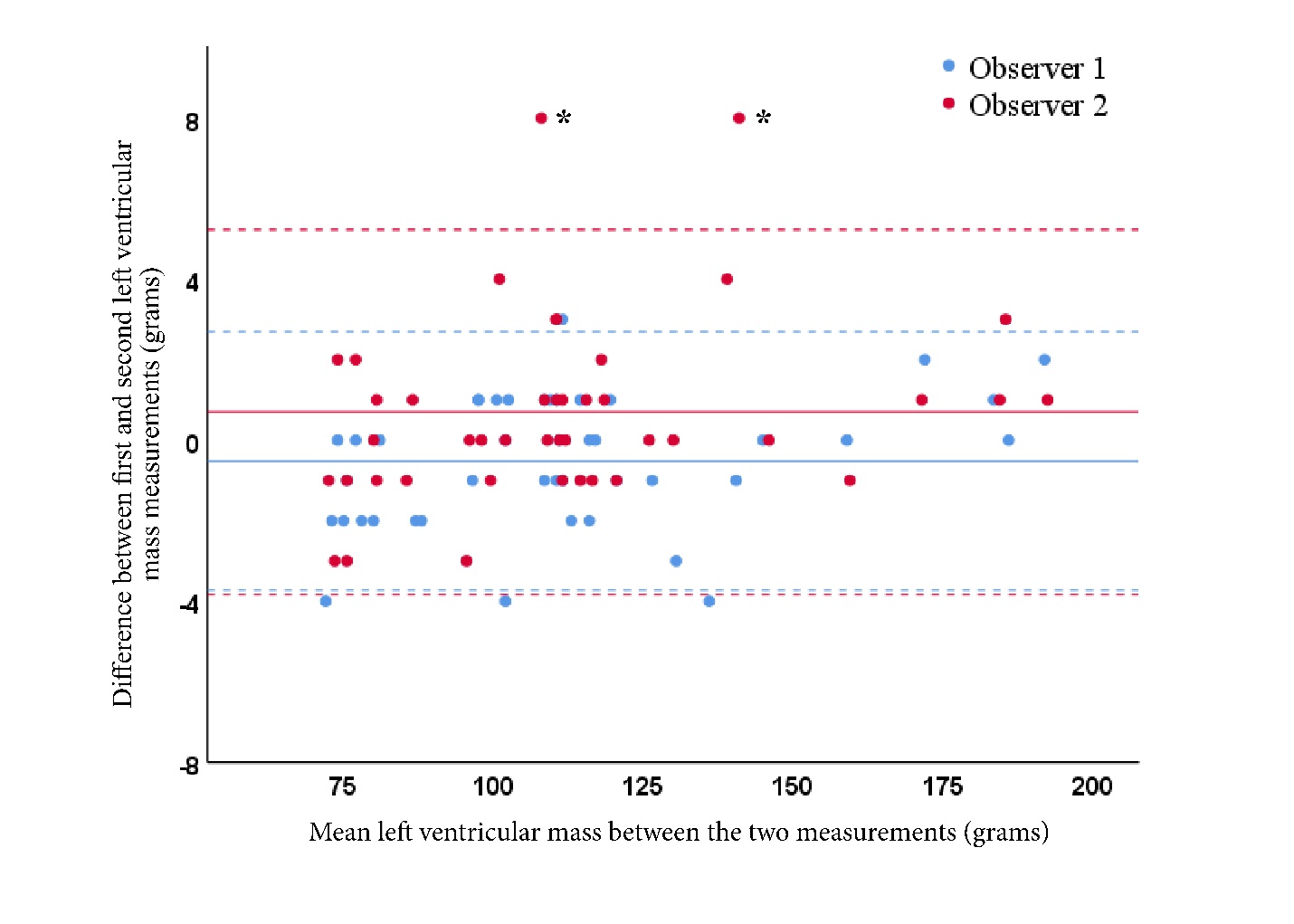


Intra-observer Bland-Altman plot for the mean value between the first and second observation of each observer (x-axis) and the LV mass differences between the first and second observation of each observer (y-axis). The solid line represents the mean of all measurements paired per observer. Both observer’s measurements have their own 95% confidence interval limits of agreement, shown by the dotted lines. The variability for observer 2 is slightly higher than the variability for observer 1, likely attributed to the two cases with outlying difference values around 8, marked with an asterisk.

**Supplementary Figure 2** - Inter-observer agreement Bland-Altman plot

*
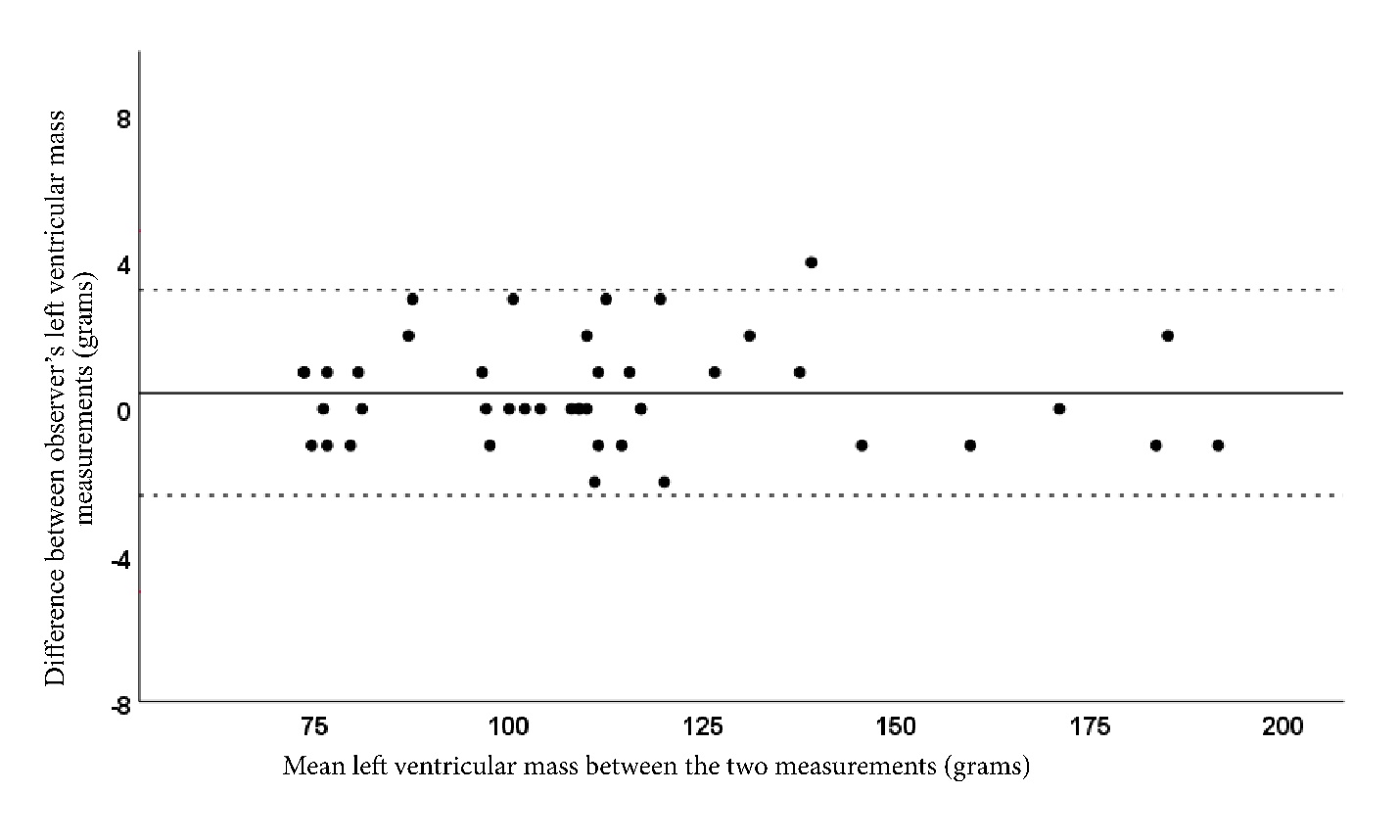
*

Interobserver Bland-Altman plot for the mean value between the two observer’s LV mass measurements (x-axis) and the LV mass differences between the two observer’s observations (y-axis). The solid black line represents the mean of all measurements paired. The dotted lines represent the 95% confidence interval limits
